# Mechanisms for Cell-to-Cell Transmission of HIV-1

**DOI:** 10.3389/fimmu.2018.00260

**Published:** 2018-02-19

**Authors:** Lucie Bracq, Maorong Xie, Serge Benichou, Jérôme Bouchet

**Affiliations:** ^1^Inserm U1016, Institut Cochin, Paris, France; ^2^CNRS, UMR8104, Paris, France; ^3^Université Paris-Descartes, Sorbonne Paris-Cité, Paris, France; ^4^International Associated Laboratory (LIA VirHost), Institut Pasteur Shanghai-Chinese Academy of Sciences, Shanghai, China; ^5^International Associated Laboratory (LIA VirHost), CNRS, Université Paris-Descartes, Institut Pasteur, Paris, France

**Keywords:** HIV-1, cell-to-cell transfer, macrophages, dendritic cells, T cells

## Abstract

While HIV-1 infection of target cells with cell-free viral particles has been largely documented, intercellular transmission through direct cell-to-cell contact may be a predominant mode of propagation in host. To spread, HIV-1 infects cells of the immune system and takes advantage of their specific particularities and functions. Subversion of intercellular communication allows to improve HIV-1 replication through a multiplicity of intercellular structures and membrane protrusions, like tunneling nanotubes, filopodia, or lamellipodia-like structures involved in the formation of the virological synapse. Other features of immune cells, like the immunological synapse or the phagocytosis of infected cells are hijacked by HIV-1 and used as gateways to infect target cells. Finally, HIV-1 reuses its fusogenic capacity to provoke fusion between infected donor cells and target cells, and to form infected syncytia with high capacity of viral production and improved capacities of motility or survival. All these modes of cell-to-cell transfer are now considered as viral mechanisms to escape immune system and antiretroviral therapies, and could be involved in the establishment of persistent virus reservoirs in different host tissues.

## Cell-Free and Cell-to-Cell Infection

The worldwide pandemic of HIV-1 infection is a global and major public health problem, and sexual transmission is the major route of infection of the newly HIV-1-infected adults. Even though the use of antiretroviral therapies led to a net decrease of morbidity and mortality of HIV-1-infected patients, a better understanding of the cellular mechanisms involved in the sexual transmission and early dissemination of the virus is still required in order to rationally design more specific and potent strategies for HIV-1 prevention. A research priority for HIV-1 eradication is then the elucidation of the events involved in the mucosal viral transmission through the mucosa of the male and female rectal and genital tracts. While both cell-free and cell-associated HIV-1 are present in the semen, vaginal secretions, and anal mucus, and are thought to contribute to the virus sexual transmission, the cell-associated HIV-1 mucosal transmission has been largely understudied. Cell-to-cell transmission of HIV-1 involves various cell types of the immune system, including T lymphocytes, macrophages, and dendritic cells (DCs). Whereas the infection of T lymphocytes *via* cell-to-cell transfer was broadly investigated *in vitro*, there is a paucity in knowledge of the mechanisms that control infection and virus dissemination in macrophages and DCs by cell-to-cell transfer. Yet, macrophages and DCs play crucial roles in the physiopathology of infection, with macrophages being involved in the establishment of persistent virus reservoirs in different host tissues while DCs participate in the early stages of virus transmission and dissemination after primo-infection at the level of genital and rectal mucosa. Specific therapies blocking HIV-1 mucosal transmission to these target cells should therefore be designed.

While HIV-1 infection of target cells with cell-free viral particles has been largely documented, pioneer studies from early 1990s showed that HIV-1 dissemination was largely increased through the establishment of direct cell-to-cell contacts between infected donor CD4+ T cells and target T cells [for review: Ref. (1)]. At least *in vitro*, cell-to-cell transfer of HIV-1 between T cells leads to a massive and very efficient infection that may be 100–1,000-fold more efficient than infection carried out by cell-free viral particles (1–5). The efficiency of cell-to-cell infection between CD4+ T cells has been related to a high-multiplicity of infection at the site of the cell–cell contact, probably leading to the integration, and accelerated viral gene expression of multiple proviruses in the target cell (6–10). While some reports suggest that cell-to-cell infection could be the main route of HIV-1 infection *in vivo* (10, 11), the specific contribution of cell-free and cell-to-cell infection by HIV-1 in infected hosts is still a matter of debate. Using multiphoton intravital microscopy in HIV-1-infected “humanized” mice, Murooka et al. showed that HIV-1-infected T cells establish interaction with surrounding cells and can even form syncytia with other lymph node-resident cells. The potency of infected T cells in lymph nodes to migrate may facilitate virus cell-to-cell transmission and spreading *in vivo* (12). Interestingly, exposure of human or macaque mucosal explants to HIV-1- or SIV-infected cells, allows more efficient viral transmission and infection than cell-free viruses (13, 14), suggesting the potency of HIV-1- or SIV-infected T cells to transmit viruses and propagate infection in host tissues. The high efficiency of cell-to-cell infection was also proposed to be a mechanism for HIV-1 to escape to antiretroviral therapy and neutralizing antibodies (15) but these results are still controversial and will be discussed below (4, 6, 16).

Different modes of infection through different cellular structures enabling close contacts between virus-donor cells and recipient target cells have been described over the past years for cell-to-cell transmission of HIV-1 *in vitro*. Intercellular transfer of viral material has been described mainly through establishment of the infectious or virological synapses, but also using membrane protrusions such as filopodia and tunneling nanotubes (TNTs), and cell fusion or cell engulfment processes. The implication of these different structures and processes for cell-to-cell transfer and dissemination of HIV will be discussed in this review.

## Nanotubes, Filopodia

The role of membrane protrusions in intercellular communications has been widely explored [for review: Ref. (17)]. A large diversity of membrane protrusions have been described, both *in vitro* (18, 19) and *in vivo* (20–22), and play important roles in the transmission of information between cells from different physiological systems, such as neurons (18, 23, 24), myeloid cells (25–29), or T cells (30).

Among the described membrane protrusions, two different types of nanotubes have been reported, corresponding to close-ended nanotubes and open-ended nanotubes (also known as TNTs) (27, 31, 32). Intercellular communications involving TNTs were first observed in 2004 as F-actin-containing membrane extensions able to connect distant cells during minutes to hours (18). TNTs are fragile and dynamic structures extended up to 100 µm in length with diameters ranging from 50 to 200 nm, and are not attached to the substratum (18, 30). They can mediate and facilitate the transfer, between several cell types, of cytoplasmic, and plasma membrane molecules, Ca2+ (29, 33), cargos including vesicles derived from various organelles such as early endosomes, endoplasmic reticulum, Golgi complex, and lysosomes (24, 33, 34), and even bigger cellular organelles like mitochondria and endosome-related structures (18, 32), but also pathogens such as bacteria (28).

**Figure 1:**
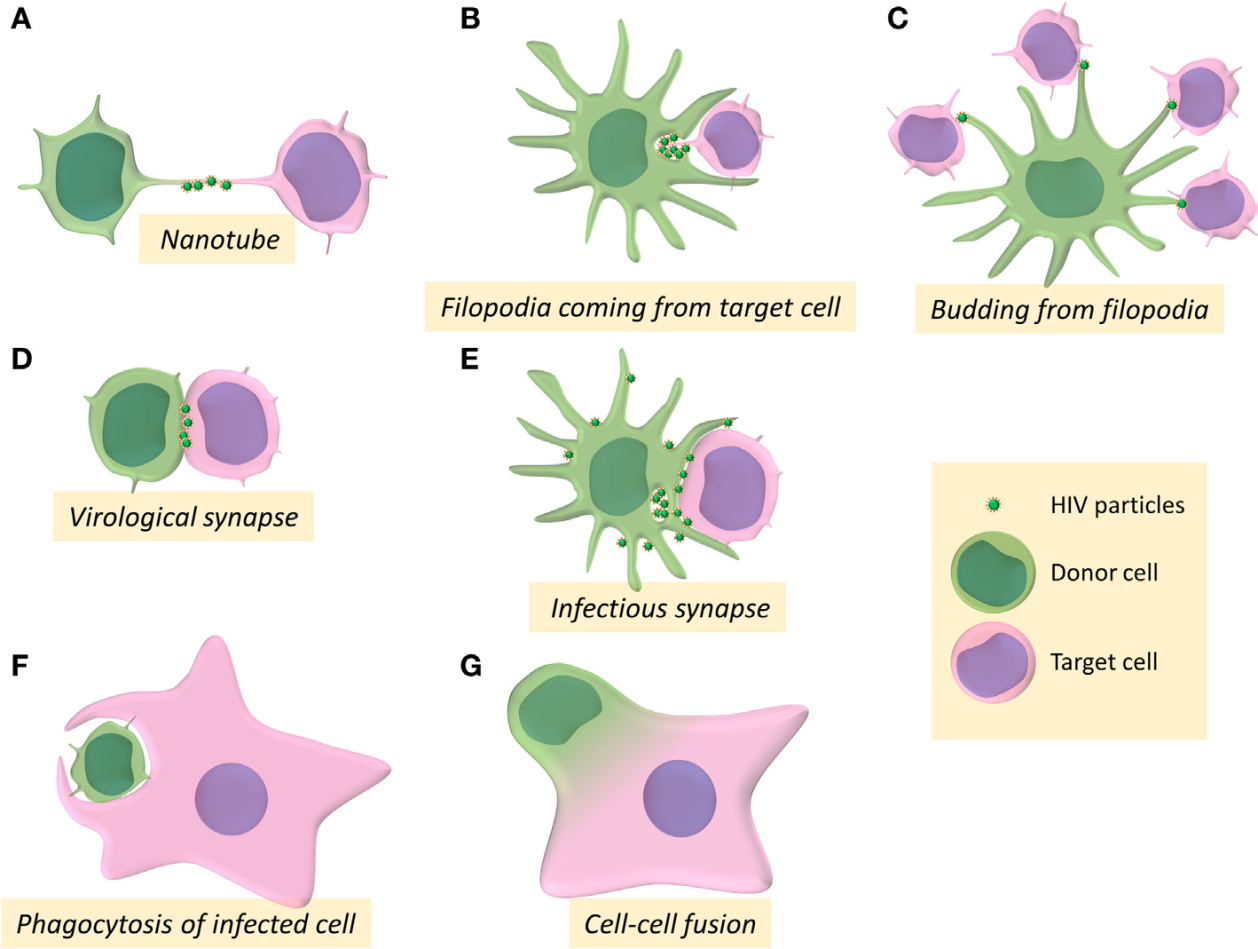
Intercellular structures and processes involved in cell-to-cell transmission of HIV-1. **(A–G)** Schemes represent the different pathways for HIV-1 cell-to-cell transfer between donor cells (in green) and target cells (in pink).

Several studies showed that HIV-1 utilizes TNT networks to move from one cell to another leading to virus cell-to-cell transfer (25, 30, 34, 35) (Figure 1A). The frequency of TNT formation is not affected by HIV-1 in T cells but these structures could allow rapid spread of virus between T cells (30). Virus particles can thus be transferred by surfing along the surface of TNTs between T cells (30). Virus dissemination through TNTs was also reported between macrophages, in which HIV-1 particles can be transferred through intracellular vesicles derived from the endosomal reticulum or the Golgi apparatus (34, 35). Furthermore, in macrophages, HIV-1 increases the number of these intercellular structures to infect new cells (25). The HIV-1 Nef auxiliary protein has been reported to be responsible for the formation of TNTs in the THP-1 macrophage-like cell line (36) as well as in primary monocyte-derived macrophages, in which Nef alters the localization of the scaffolding protein M-Sec (37), which is a key regulator of TNT formation by a still undefined mechanism (26).

Another route of viral cell-to-cell transmission through membrane extension involving formation of filopodia has been first described for transmission of the retroviral murine leukemia virus (MLV) (19). Filopodia are F-actin-rich thin plasma membrane extensions that are involved in several cellular functions, such as chemo-migration, adhesion to the extracellular matrix, or formation of cell–cell contacts [for review: Ref. (38)]. In DCs, after engagement of the lectin DC-SIGN, HIV-1 mediates the activation of the small GTPase CDC42 and the remodeling of actin cytoskeleton to promote filopodia extension that allows virus transmission to neighboring CD4+ T cells (39) (Figure 1B). By budding at the tip of filopodia in DCs, HIV-1 could be able to tether concomitantly several neighboring CD4+ T cells, leading to viral transfer and infection of the target T cells (40) (Figure 1C).

## The Virological Synapses

The formation of the so-called virological synapse is the major and well-established route for viral cell-to-cell transmission, and was first described in the context of human T-lympho-tropic virus infection as a close and organized cell-to-cell contact structure between an infected donor cell and a target cell, enabling the transfer of viral material between the two cells (41) (Figure 1D). The virological synapse has been named after its homologies with the immunological synapse formed between antigen-presenting cells (APCs) and T cells for antigen presentation. During the formation of the immunological synapse, binding of the T-cell receptor (TCR) to the MHC-peptide complex expressed at the surface of APCs ensures T-cell activation by transducing signals that cause transcriptional upregulation of numerous genes, polarized secretion of cytokines or cytotoxic granules, and cell-proliferation (42, 43). Microtubules and actin cytoskeleton, together with adhesion molecules (LFA-1 and its ligand ICAM-1), participate in the stabilization of the immunological synapse.

Whilst virus cell-to-cell transmission was defined, from the early 1990s, to be more efficient than cell-free virus infection (1, 44–46), the concept of “virological synapse” between HIV-1-infected donor cells and target cells was defined by the group of Quentin Sattentau as a “cytoskeleton-dependent, stable adhesive junction, across which virus is transmitted by directed transfer” (47). The virological synapse shares several common features with the immunological synapse. Indeed, formation of both virological and immunological synapses involves the recruitment of receptors and cell adhesion molecules to an adhesive interface in an actin-dependent manner (48, 49). We focus here on the structure of the virological synapse established between a donor infected CD4+ T-cell and a CD4+ T-cell target which has been the best documented (48, 50, 51). The specific synapses formed between DCs or macrophages and target CD4+ T cells will be described below.

**Figure 2:**
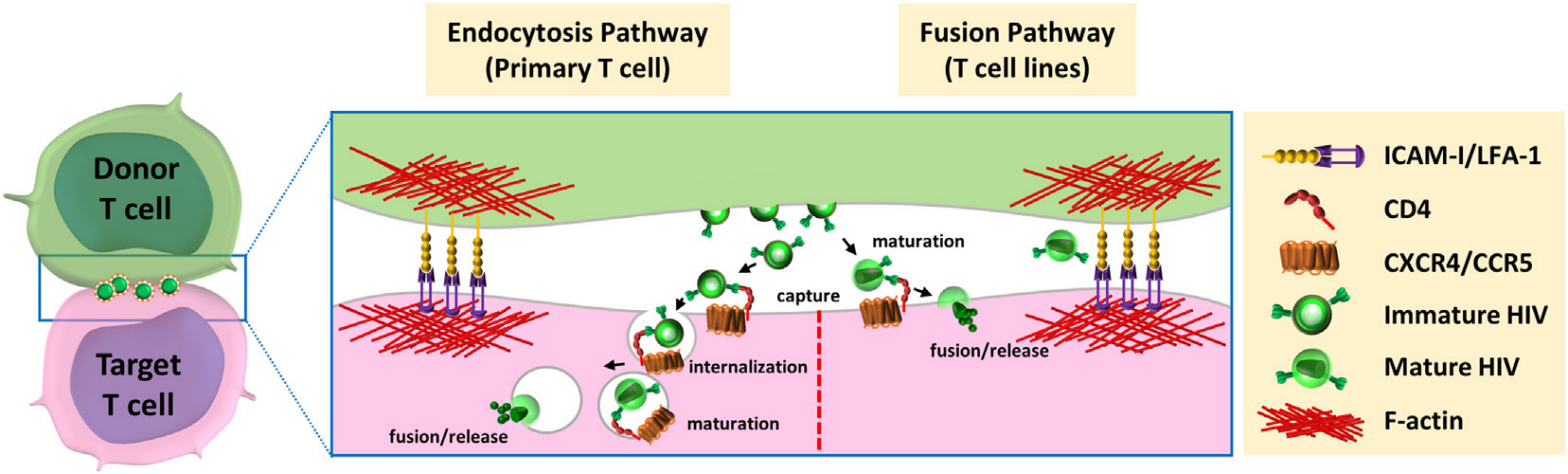
Models for HIV-1 entry downstream of the virological synapse. After budding and release from infected donor cells, HIV could be able to enter the target cells *via* two distinct pathways. (left) Entry through endocytic pathway: immature virions interact with the CD4 receptor at cell surface of target cell, are internalized by endocytosis, mature in endocytic compartments, and fuse with the luminal membrane of endosomes. (right) Fusion with plasma membrane: mature viruses are captured by CD4, at the surface of target cells, and directly fuse with plasma membrane for release of viral material in the cytoplasm of the target cells.

The virological synapse is a dynamic structure initiated by the recognition of the target T-cell surface receptor CD4 by the viral surface envelope glycoprotein gp120 expressed at the surface of the infected donor T cell (Figure 2). This interaction allows the recruitment of the viral Gag polyprotein precursor to the intercellular interface (52) and triggers the recruitment of co-receptors, CXCR4 or CCR5, adhesion molecules LFA-1, ICAM-1, and other cell surface proteins such as tetraspanins to the site of intercellular contact, for stabilization of the virological synapse and efficient viral transfer (48, 52, 53). The interaction between HIV-1 envelope glycoprotein and the receptor CD4 is the major determinant for virus transfer across the virological synapse (54). In addition to CD4, the implication of the co-receptors CCR5 and CXCR4 has been investigated by several groups. Some initial studies suggested that the formation of the virological synapse could be independent of the co-receptor usage since the inhibition of these co-receptors did not affect the number of cellular conjugates between cells (55) or HIV-1 transfer (4, 56). However, other reports showed that expression of these co-receptors was necessary for productive infection of the target cells downstream of the formation of the virological synapse (57–60). Therefore, the use of the co-receptor could be dispensable for the formation of the virological synapse but could be required for efficient infection of the target cell after viral transfer through the virological synapse.

Similarly, the role of the adhesion molecules LFA-1 and ICAM-1 in the formation of the virological synapse was debated. Initially, interaction between LFA-1 and ICAM-1 has been proposed to stabilize the virological synapse for efficient viral transfer. Jolly et al. first showed that antibodies against LFA-1 and ICAM-1 were able to partially block viral transfer through the virological synapse (48, 52). However, the percentages of inhibition were very different depending on the antibody used (40–90% inhibition using LFA-1 antibodies and 30% inhibition with ICAM-1 antibodies). The role of adhesion molecules for the formation of the virological synapse and virus transfer was then confirmed, showing a significant threefold inhibition in virus transfer using a Jurkat T-cell line lacking the α subunit of LFA-1 (61). However, a third group obtained opposite results showing that virus transfer through the virological synapse between T cells did not require LFA-1 binding to ICAM-1 (55). Using antibodies blocking adhesion molecules such as LFA-1, ICAM-1, and ICAM-3 in MOLT or primary T cells, or 293 T cells lacking LFA-1, no inhibition of viral transfer through the virological synapse was observed in this study. The different assays used to analyze viral transfer and viral production could explain this divergence. In these studies, antibodies targeting different epitopes were used to block adhesion molecules and could have differential effects on virological synapse formation and HIV-1 transfer. The discrepancy in the results obtained by several groups could also be explained by the different cell models used in these experiments. Indeed, the level of adhesion molecule expression (i.e., ICAMs and Integrins) largely differs from T-cell lines to primary T cells (52, 55). To conclude, while the recruitment of adhesion molecules to the virological synapse is well established (52, 53), their specific role and requirement for cell-to-cell transmission of HIV-1 remains to be determined.

Finally, specific rearrangements of the cytoskeleton are required for the formation of the virological synapse and efficient viral transfer. Engagement of the CD4 receptor by the gp120 viral envelope glycoprotein triggers actin cytoskeleton remodeling and microtubule polarization toward the virological synapse (51). These cytoskeleton modifications are needed for polarization of the Gag precursor and envelope glycoproteins to the site of cell–cell contact (61, 62). By comparison, engagement of the TCR by MHC-antigen complex expressed at the surface of APC induces a polarization of the T cell toward the immunological synapse that triggers a cascade of intracellular signals leading to cytoskeleton remodeling, cytokine gene expression, proliferation, and execution of the T-cell effector functions. Similarly, polarization of the microtubule-organizing center (MTOC) of infected donor T cells toward the virological synapse has been observed in 30–60% of the conjugated formed with target T cells (54, 63, 64). Viral envelope glycoproteins are also reoriented to the virological synapse through their interaction with an intracellular compartment associated with the polarized MTOC. This suggests an active role of the microtubule network in the recruitment of the viral envelope to the virological synapse (4, 53, 54, 59, 63). Electron microscopy analyses with tomography reconstruction revealed the polarization of organelles such as mitochondria, lysosomes at the site of cell–cell contact in around 75% of the virological synapse formed. The colocalization of viral material with secretory lysosomal compartments and the relocalization of these vesicular compartments toward the cell–cell interface indicates that HIV-1 could take advantage of intracellular traffic and secretory pathways to disseminate through the virological synapse (63). Some molecules involved in T-cell signaling also participate in cell-to-cell transmission of HIV-1. Accordingly, polarization of the cells can be mediated by ICAM-1/LFA-1 signaling, which in addition to its effect on stabilizing virological synapse formation, induces a ZAP70-dependant signaling pathway for cytoskeleton remodeling, T cells polarization, and efficient HIV-1 transfer at the virological synapse (53, 64). In target T cells, CD4 engagement by viral gp120 induces phosphorylation of the T-cell specific Src kinase Lck allowing the recruitment of several TCR signaling molecules such as ZAP70, LAT, SLP76, or PLCγ in their active phosphorylated form. This unusual signaling does not trigger regular T-cell activation nor proliferation, but could induce a local depletion of F-actin at the center of the virological synapse that could facilitate viral transfer to target cells (54).

The first observation of the virological synapse by electron microscopy by Jolly et al. showed that mature HIV-1 particles were clustered in the synaptic space (48), but they did not show evidence of endocytosis of viral particles by target cells, suggesting that mature viruses released from the donor T cells at the virological synapse space could fuse directly with the plasma membrane of the target T cells (48). Another group also observed HIV-1 particles in the synaptic space by electron microscopy, but suggested that these particles could be internalized by target cells into trypsin-resistant large intracellular vesicles containing several virus particles. This internalization of viruses could occur using an actin-dependent mechanism through lamellipodia-like structures (56). Several groups confirmed that HIV-1 can be internalized by endocytosis at the virological synapse. Therefore, immature HIV-1 particles have been shown to be transferred across the virological synapse through dynamin- and clathrin-dependent endocytosis leading to productive infection of the target T cells (59, 65–67). After internalization, the HIV-1 particles were found in intracellular compartments that co-localized with the early-endosomal marker EEA1 but not with the lysosomal-associated membrane protein LAMP1, indicating that viruses are internalized in endosomal compartments but are not addressed to lysosomal degradation (68). In accordance with these results, Dale et al. demonstrated that after endocytosis of immature virions by the target T cells, the cleavage of the Gag polyprotein precursor by the viral protease induced the maturation of the viral particle in some endosomal compartments (57). This cleavage restored the membrane fusion activity of the viral envelope and led to viral-cell membrane fusion into these endosomal compartments.

However, the group of Quentin Sattentau, who first described the formation of the virological synapse for HIV-1 cell-to-cell transfer, failed to observe endocytosis of viral particles at the virological synapse using confocal microscopy, electron microscopy, or electron microscopy coupled with tomography (47, 60). Puigdomenech et al. hypothesized that these different results could be explained by the different experimental systems used and the use of primary T cells or immortalized T-cell lines (69). They suggest that these differences may be associated with the different kinetics of the fusion events observed between primary CD4+ T cells and T-cell lines. Delayed fusion at the plasma membrane of the target cell may increase virus endocytosis in primary cells (Figure 2, left). In contrast, a rapid fusion at the cell membrane in T-cell lines may favor HIV-1 transmission without need for endocytosis (Figure 2, right). From this hypothesis, these authors suggest that endocytosis of virus particles is the main mechanism used by HIV-1 for cell-to-cell transfer and infection of primary CD4+ T cells. Remarkable differences between virological synapses formed with Jurkat cells or primary CD4+ T cells have been indeed reported (70). Scanning electron microscopy experiments revealed strong differences in spatial distribution of virions at the intercellular interface and important differences in the architecture of the contacts between T cells. These differences could be due to the increase deformability of primary T cells compared with the immortalized Jurkat cells, and questioned the relevance of cell line models used so far for studying virological synapse formation and cell-to-cell transmission of HIV-1.

It is noteworthy to mention that cell-to-cell transfer of HIV-1 between T cells is associated to an increase mortality of the target cells, when compared with cell-free infection of T cells. First, the massive entry of viral particles into target cells across the virological synapse has been shown to be responsible for caspase-1-mediated pyropoptosis of target T cells (71). In addition, while the formation of the virological synapse is triggered by gp120/CD4 interaction, it has been reported that upon cell-to-cell contact, the gp41 transmembrane viral glycoprotein was able to trigger hemifusion but not fusion events responsible for rapid CD4+ T-cell death (72, 73).

### Viral Transfer and Resistance to Neutralizing Antibodies and Antiretroviral Drugs

Like some other viruses (Herpesviruses, poxviruses, and Hepatitis C virus), it has been proposed that HIV-1 could escape, at least partially, to neutralization by specific antibodies targeting the viral envelope when it is transferred across the virological synapse. Failure of inhibition of HIV-1 cell-to-cell infection by neutralizing antibodies was first suggested in 1995 in a study showing that antibodies against the glycan V3-loop of gp120 were unable to block cell-to-cell viral transfer (74). However, after characterization of the virological synapse for HIV-1 cell-to-cell transfer in 2004, several different groups tried to elucidate the mechanisms of neutralizing antibody escape in the virological synapse-mediated viral transfer. There is a general agreement that the potency of neutralizing antibodies is reduced during cell-to-cell transmission compared with cell-free infection and that only a subset of neutralizing antibodies can efficiently inhibit cell-to-cell transmission (47, 56, 75–77). For example, it was reported that several specific anti-gp120 antibodies targeting the CD4 binding site lost considerable potency (10- to 100-fold decrease) when HIV-1 was transferred by cell-to-cell transmission (75). Though some antibodies are still able to block cell-to-cell viral transmission at high concentration, VRC01, which is one of the most potent antibody for inhibition of cell-free infection, is particularly ineffective for blocking cell-to-cell viral transfer (75). While most anti-gp120-directed antibodies, and in particular those directed against the gp120 CD4 binding site displayed a reduced activity during viral cell-to-cell transmission, the same group reported that the T20 fusion inhibitor targeting the gp41 transmembrane glycoprotein as well as neutralizing antibodies directed against gp41 maintained their activity and were thus able to block both cell-free and cell-to-cell infection with the same efficiency. However, other groups showed that some anti-gp41 neutralizing antibodies failed to inhibit cell-to-cell transmission (4, 56, 77). Globally, the efficiency of neutralizing antibodies for neutralization of the virological synape-mediated viral transfer is variable and some epitopes of the viral envelope glycoproteins seem more susceptible than others to neutralization of the viral cell-to-cell transfer between T cells.

Similarly, the activity of the T20 peptide entry inhibitor of the Env-mediated membrane fusion on HIV-1 transmission through the virological synapse is still a matter of debate, and a lot of contradictory results have been published regarding the effect of this inhibitor. Whereas it was initially reported that T20 was unable to block virological synapse-mediated viral transfer using flow cytometry analysis (4), Martin et al. then showed that cell-free and cell-to-cell infection across the virological synapse were equivalently susceptible to T20, using qPCR for detection of infection in the target T cells (60). These different results can be explained by the different experimental approaches used since the first study was looking for viral transfer of viral material when the other study analyzed *de novo* synthesized viral DNA after viral transfer. We can hypothesize that the T20 fusion inhibitor does not affect viral transfer across the virological synapse, but inhibits HIV-1 infection in the target cell after the viral transfer mediated through the virological synapse. The fusion inhibitors T20 and C34, targeting gp41, could also have no effect on capture and endocytosis of virus particles for viral cell-to-cell transfer, but would rather block subsequent viral fusion in the endosomal compartments leading to inhibition of productive infection (65).

While it is usually accepted that HIV-1 could escape or is less sensitive to the inhibitory activity of antiviral drugs used in clinic when T cells are infected by virus transfer through the virological synapse, only a few studies have reported rational analyses of the effects of antiretroviral drugs such as anti-protease or anti-reverse transcriptase inhibitors on viral cell-to-cell transfer. Some studies indeed reported that HIV-1 virological synapse-mediated infection and cell-free infection were similarly inhibited by anti-protease drugs (15, 78). Regarding reverse transcriptase inhibitors, it seems that non-nucleoside-analog inhibitors of the reverse transcriptase could block virological synapse-mediated infection of T-cell targets (15, 78), whereas nucleoside-analog reverse transcriptase inhibitors (NRTI) were unable to do it. However, it was also reported that cell-to-cell infection was blocked efficiently when these NRTIs were used in combination even if the level of inhibition is lower than for cell-free infection (15, 78, 79). Usage of HIV-1 protease inhibitors Lopinavir and Darunavir has been reported as an efficient way to inhibit cell-to-cell transmission as efficiently as cell-free infection (79). This effect of protease inhibitors compared with reverse transcriptase inhibitors is suspected to be due to their ability to target immature virions and blocking their maturation in fully infectious viruses.

In conclusion, it seems from the data reported in the literature that the idea that the cell-to-cell viral transfer through the virological synapse can escape from neutralizing antibodies and antiretroviral drugs is not so evident and is probably dependent on the inhibitors used. Further systematic investigations with standardized assays to evaluate and compare activities of neutralizing antibodies and antiretroviral drugs in both cell-free and cell-to-cell infections are absolutely needed to address this important point.

## Heterogeneity of the Virological Synapses

HIV-1 transmission through the virological synapse established between an infected donor T cell and a recipient target T cell have been extensively studied. Nonetheless, DCs and macrophages can also transmit HIV-1 to target CD4+ T cells through the formation of a related virological synapse (80–82). If virus cell-to-cell transfer between DCs and T cells, as well as between macrophages and T cells, presents some similarities with the virological synapse observed between T cells, they also showed some specific differences.

### The DC Infectious Synapse

Regarding DCs as virus-donor cells, two types of cell-to-cell viral transfer and infection of T-cell targets have been proposed: in *cis*-infection, DCs are productively infected and can then transfer viruses to CD4+ T cells, whereas in *trans*-infection, DCs are able to capture HIV-1 independently of CD4 and then transfer viruses to CD4+ T cells through the formation of the so-called “infectious synapse” (39, 83–85) (Figure 1E). HIV-1 *cis*-infection and *trans*-infection of CD4+ T target cells from DCs have been proposed to be mediated by distinct processes (86). However, because DCs highly express antiviral cellular restriction factors (APOBEC3G, TRIM5α, BST-2, and SAMHD-I), HIV-1 replicates poorly in this cell type. That is probably the reason why most of the studies of cell-to-cell transmission of HIV-1 from infected DCs to T cells developed *cis*-infection models of DCs using massive quantities of VSV-G-pseudotyped HIV-1 particles to infect DCs *in vitro*, thus questioning the validity of these models of *cis*-infection.

Regarding the *trans*-infection cell-to-cell process from DCs, several studies showed that DCs can capture HIV-1 through binding of the viral envelope glycoproteins to the mannose specific C-type lectin receptor (DC-SIGN) and store viruses into tetraspanin-enriched compartments, in continuity with the plasma membrane without viral replication in recipient DCs (58, 83, 84, 87). More recently, it has been reported that the immunoglobulin(I)-type lectin Siglec-1 (or CD169) can also bind to HIV-1 particles carrying sialyl-lactose gangliosides on their envelope (88–90). After capture, viruses remain at the cell surface or are internalized in vesicular containing compartments (see below). Then, DCs are able to transfer these viruses to CD4+ T cells independently of viral replication through the formation of an “infectious” synapse (91–94), thus decreasing the efficiency of broadly neutralizing antibodies on HIV-1 infection of target T cells (95).

While the formation of the virological synapse depends on the interaction between the viral gp120 envelope glycoprotein and the CD4 receptor, the infectious synapse formed during *trans*-infection does not rely upon CD4/gp120 interaction since Rodriguez-Plata et al. showed that the number of cell-to-cell conjugates formed between DCs and CD4+ T cells was not increased in the presence of HIV-1 (85). However, they also reported that the formation of the infectious synapse was decreased by 60% when the interaction between the adhesion molecules ICAM-1 and LFA-1 was disrupted. Furthermore, recognition of MHC-superantigen complexes presented at the surface of DCs by the TCR significantly enhanced virus transfer across the infectious synapse (85, 91). These authors suggest that the formation of the infectious synapse is not triggered by the virus but is more related to a hijacking of the immunological synapse by HIV-1. However, their experiments do not prove formally the formation of a canonical immunological synapse in their system as TCR or signaling molecules recruitment at the interface was not verified. Moreover, a previous study failed to detect any MHC-II or TCR recruitment at the interface between DCs carrying viruses and target CD4+ T cells, thus excluding the identification of this structure as a *bone fide* immunological synapse (87).

While the formation of the infectious synapse does not rely on CD4/gp120 interaction, this interaction is still required for efficient productive infection of CD4+ T cells following formation of cell-to-cell conjugates with DCs carrying HIV-1. After the formation of conjugates, the CD4 receptor and CXCR4 or CCR5 co-receptors are recruited at the site of the cell–cell contact (96). Furthermore, intercellular transfer to CD4+ T cells of virus particles captured by DCs can be blocked using high concentrations of broadly neutralizing antibodies targeting the gp120 and gp41 envelope glycoproteins (97).

Rearrangements of the actin cytoskeleton also play a key role in HIV-1 transfer across the infectious synapse. A recent screening shows that tetraspanin 7 and dynamin-2 control nucleation and cortical stabilization of actin to maintain viruses onto dendrites for efficient cell-to-cell transfer from DCs to CD4+ T cells when cells are cocultured, and simultaneously infected with HIV-1 (98). Furthermore, it has been shown that large sheet-like membrane structures derived from DCs carrying HIV-1 wrap around T cells leading to a large interface at the infectious synapse (58). Within this interface, filopodia extensions from T cells are able to interact with the virus-containing compartments (VCCs) in continuity with the plasma membrane of DCs for efficient cell-to-cell transmission (58). Together, these results show that in DCs, HIV-1 can be efficiently transferred by hijacking the immunological synapse without productive infection of DCs.

As specialized DCs present in cervico-vaginal and rectal mucosa, Langerhans cells have been proposed to be the first targets of HIV-1 after sexual exposure. However, these cells were proposed to capture and degrade captured virions through expression of the DC-SIGN-related lectin Langerin at their surface, avoiding virus transmission from Langerhans cells to CD4+ T cells (99). More recently, in an *ex vivo* human tissue explant model, Ballweber et al. showed that vaginal Langerhans cells were able to transmit HIV-1 to CD4+ T cells, without being productively infected, rendering HIV-1 particles insensitive to inhibition by reverse transcriptase inhibitors in this context (100).

Of note, some studies suggest that the expression of Siglec-1 in macrophages mediates the internalization of HIV-1 particles in a VCC. As for DCs-to-T-cell transmission, the presence of these storage compartments in macrophages allows for the transfer of virions to CD4+ T cells (3, 101). Interestingly, the role of Siglec-1 for viral capture by macrophages has been evidenced *in vivo* in lymph node from HIV-1 or MLV infected mice (90). By showing subsequent transfer of MLV from macrophages to B cells, Sewald et al. proposed that this mechanism could be transposed to HIV-1 macrophage-to-T-cell transmission (90).

### HIV-1 Transfer through Conjugates Formed with Macrophages

Compared with the cell-to-cell transmission mechanisms of HIV-1 involving T cells or DCs as infected donor cells, little is known about the formation of conjugates between HIV-1-infected macrophages and target CD4+ T cells. As evidenced by the presence of infected macrophages in different tissues in infected patients, macrophages are cellular targets of HIV-1 and probably play an important role in HIV-1 pathogenesis (102–108). Like other infected cells, they participate in cell-to-cell transmission of HIV-1 and are essential for HIV-1 spread in tissues of infected patients. One major feature of HIV-1-infected macrophages is related to the assembly and budding steps of the new viral particles. While in T cells viral assembly takes place at the plasma membrane, *de novo* formed viral particles accumulate both at the plasma membrane and in tetraspanin-enriched compartments called VCCs in productively infected macrophages (109–112). These VCCs, which do not exhibit the same characteristics than endosomes or multivesicular bodies, but are characterized by the presence of the tetraspanins CD63 and CD81 (113–116), and are connected to the extracellular space by thin channels in continuity with the plasma membrane (110, 117, 118). Viruses thus assemble in macrophages in a protected compartment and are then released in the extracellular environment.

Infected macrophages can efficiently transfer viruses to uninfected CD4+ T cells across the formation of a virological synapse but the mechanisms involved in the formation of this macrophage/T-cell virological synapse are not completely characterized (3, 119). During the formation of the conjugates between infected macrophages and target T cells, VCCs could rapidly move to the virological synapse (82) through an actin cytoskeleton-dependent mechanism (81). Similarly to the virological synapse formed between T cells, the virological synapse involving virus-donor macrophages leads to the recruitment of CD4, CCR5, LFA-1, and ICAM-1, as well as the viral Gag precursor and envelope glycoproteins at the site of cell–cell contact (81, 119). In contrast to the virological synapse formed between T cells, which is dependent of CD4, the formation of conjugates between infected macrophages and CD4+ T cells appears to be independent of gp120 (82), while the viral transfer is dependent of gp120/CD4 and LFA-1/ICAM-1 interactions (81). Through the formation of the virological synapse, infected macrophages can transfer a high multiplicity of HIV-1 to CD4+ T cells, promoting reduced viral sensitivity to reverse transcriptase inhibitors as well as to a panel of neutralizing antibodies (81, 82). While the virological synapse between infected macrophage and T-cell targets show several differences with the virological synapse between T cells (82, 119, 120), no similar structure has been described so far, to our knowledge, between infected T cells and uninfected macrophages for virus cell-to-cell transfer in these HIV-1 target cells.

## Engulfment of Infected T Cells by Macrophages and Entosis

Despite the fact that HIV-1 infection of T cells by cell-to-cell transfer has been largely documented, cell-to-cell infection of macrophages remains poorly investigated. Recently, the group of Quentin Sattentau pointed out for the first time a new mechanism of specific cell-to-cell transfer of HIV-1 from infected CD4+ T cells to macrophage targets (121) (Figure 1F). The authors showed that macrophages could engulf HIV-1-infected T cells leading to productive infection of the macrophage targets. The engulfment of infected healthy but rather dying T cells was significantly higher compared with uninfected healthy T cells, indicating that the cell death and infection of virus-donor T cells might independently promotes T-cell engulfment by macrophages. This preferential uptake of infected dying T cells was independent of the interaction between gp120 and the CD4 receptor but was dependent of remodeling of the actin cytoskeleton. Since this engulfment/uptake of infected T cells by macrophages was dependent of actin remodeling and was not inhibited by amiloride, an inhibitor of macropinocytosis, the authors suggested that this engulfment process likely results from phagocytosis mechanisms.

While the uptake of infected T cell by macrophages was independent of the Env/CD4 interaction, the productive infection of macrophages following uptake of T cells would be dependent of this interaction, and also depended of the tropism of the viruses. The uptake of infected T cells was evidenced by a concentrated localization of the viral Gag precursor in the macrophage target observed in association with the CD3 T-cell specific marker. The engulfment of T cells infected with CCR5-tropic viruses led to productive infection of the macrophage targets able to produce infectious virus particles in the cell-culture supernatant. In contrast, after engulfment of T cells infected with CXCR4-tropic viruses, no productive infection of the macrophage targets was observed (121). From these data, it appears evident that macrophages can be productively infected by phagocytosis of HIV-1-infected T cells, but the mechanisms for virus entry in macrophages from intracellular phagocytosed T cells remains to be defined. Nonetheless, the dependency of co-receptor usage by HIV-1 particles for productive infection could indicate a fusion of viral particles with the phagosome membrane.

Phagocytosis of SIV-infected T cells by myeloid cells *in vivo* was also suggested by the group of Jason Brenchley in SIV-infected macaque models (122, 123). Their studies point out the presence of viral RNA and DNA originating from T cells in myeloid cells from spleen and lymph nodes of infected monkeys, but do not formally prove that this presence of specific markers was related to a phagocytosis mechanism of SIV-infected T cells by myeloid cells, since no *per se* phagocytosis experiment was carried out in these studies.

Of note, another type of engulfment of HIV-1-infected CD4+ T cells by epithelial cells *via* an entosis mechanism has been observed. Entosis refers to the invasion of non-apoptotic cells into another live cell and then induces the formation of cell-in-cell structures (124, 125). Using infected cells from an immortalized T-cell line, Ni et al. reported that epithelial cells could internalize infected T cells *in vitro*, as well as *in vivo* in sigmoid colon explants from HIV-1-infected patients showing the formation of cell-in-cell structures (126). Through this entosis process, the epithelial cells could be productively infected, as evidenced by the diffuse intracytoplasmic staining of the viral capsid protein.

## Cell–Cell Fusion

### Formation of T-Cell Syncytia

Cell-to-cell fusion between infected CD4+ T cells, or between infected and uninfected CD4+ T cells, has been initially proposed to be another mechanism for HIV-1 infection and dissemination between T cells (127–131). Early studies suggested that infected T cells could fuse with uninfected T cells to form giant syncytia, 5–100 times bigger than individual cells (131, 132). In this context, cell-to-cell fusion occurs through actin cytoskeleton rearrangements, is dependent of LFA-1/ICAM-1 interaction and is mediated through interaction between envelope glycoproteins expressed at the cell surface of infected cells and CD4 expressed on the target cells (127, 129, 131, 133). Yet, these T-cell syncytia, initially observed only *in vitro* with infected immortalized T-cell lines, have been shown to die rapidly by a mitochondrion-dependent apoptosis mechanism (134, 135). The formation of HIV-1 T cell syncytia leads to activation of the serine-threonine kinase, mTOR, which mediates phosphorylation of the transcription factor p53. Thus, p53 induces upregulation of the pro-apoptotic regulator Bax leading to mitochondrial permeabilization and release of pro-apoptotic mitochondrial proteins (136, 137). Several groups thus proposed that this cytopathic effect related to the formation of T-cell syncytia could be the mechanism of the CD4+ T cells loss observed during infection in HIV-1-infected patients (127, 128, 132).

However, formation of T-cell syncytia has been a controversial subject since other studies did not observed formation of T-cell syncytia using HIV-1-infected primary CD4+ T cells (3, 4, 11). Because no evidence of the formation of such T-cell syncytia was reported *in vivo*, it has been suggested that these giant syncytia could be *in vitro* artifacts only observed with immortalized cell lines and restricted to CXCR4-viruses (138). Viral strains were indeed initially classified as syncytia-inducing or non-syncytia inducing (NSI) strains, referring to their capacity to induce syncytia *in vitro*, and then to their ability to use either CXCR4 or CCR5 for virus entry, respectively (139). However, cells infected with NSI viruses can readily form syncytia with CCR5-positive cells, and a new classification of viral strains based on CXCR4 or CCR5-usage was adopted (140).

Interestingly, small T-cell syncytia, containing no more than five nuclei, have been then observed *in vivo* in lymph nodes from HIV-1-infected patients (141). In addition, some more recent studies using HIV-1-infected “humanized mouse models” reported the presence of motile infected syncytia in lymph nodes, smaller than those observed *in vitro* (12). These motile small T-cell syncytia can establish tethering interactions with uninfected T cells that may facilitate cell-to-cell transmission through the formation of a virological synapse (12, 142). Interestingly, these interactions between infected small syncytia and uninfected T cells do not lead to cell-to-cell fusion suggesting that this mechanism of cell fusion is finely regulated (12).

### Inhibition of T-Cell Fusion at the Virological Synapse

The numerous studies describing the formation of conjugates between infected and uninfected T cells for viral transfer across the virological synapse do not present observations of cell–cell fusion between the virus-donor T cells expressing the viral envelope glycoproteins and the target T cells expressing the CD4 receptor and CXCR4 and CCR5 co-receptors, confirming that this process is finely regulated during the formation of the virological synapse between T cells.

Since HIV-1 is a highly fusogenic virus that is preferentially transmitted by direct contact between two cells, the mechanisms inhibiting the fusion between an infected cell and a target cell deserves to be questioned. Indeed, in most of the T cell-to-T-cell transmission of HIV-1, a very few syncytia are observed compared with the formation of virological synapses (3, 4, 11). Moreover, large syncytia formed with T cells were characterized as rapid-dying cells (134, 136), which might indicate that HIV-1 has developed some mechanisms to inhibit the formation of these large T-cell syncytia to survive in infected host. As usually, it was proposed that HIV-1 took advantage of the intracellular systems displayed by its host, and used molecular mechanism to inhibit cell–cell fusion.

For example, the CD81 tetraspanin membrane protein is recruited at the site of cell–cell contact during HIV-1 cell-to-cell transmission and was proposed to be involved in the regulation of fusion (143). CD81 knockdown or inhibition with a specific antibody dramatically increased the capacity of infected T cells to fuse with surrounding non-infected cells, indicating that CD81 is a negative regulator of T cell–T cell fusion during virological synapse formation. CD81 expression at the virological synapse seems to be regulated by the actin/plasma membrane connector, ezrin. Accordingly, reduction of ezrin expression in T cells decreased the level of CD81 localized at virological synapses, thus increasing cell–cell fusion between infected T cells (144). Other tetraspanin proteins such as CD9, CD63, or CD82, have been also studied for their role in HIV-1-mediated cell–cell fusion (144, 145). In addition, it has been shown that CD63 may interact with the CXCR4 co-receptor and induces downregulation of CXCR4 expression at the cell surface, resulting in the inhibition of HIV-1 infection (146, 147). Like CD81, these molecules may act as regulators of cell fusion, but most of the experiments have been performed using HeLa or HEK293T non-lymphoid cell lines. Therefore, experiments need to be performed in more relevant cell models to precisely define the molecular requirement of these tetraspanins in the regulation of syncytia formation between HIV-1-infected T cells.

### Presence of Multinucleated Giant Cells (MGCs) in Infected Tissues

The mechanism of T-cell syncytia formation has been largely documented *in vitro* and discussed for its *in vivo* relevance. Nonetheless, processes of cell–cell fusion for HIV-1 infection and dissemination are not restricted to T cells. Some studies showed that infected multinucleated macrophages, as well as multinucleated DCs, could be found in different tissues *in vivo* in HIV-1-infected patients (148, 149). The presence of HIV-1-infected multinucleated syncytia expressing specific DC markers was found at the surface of the nasopharyngeal tonsils of HIV-1-infected patients and reported 20 years ago (148). The same group reported that infected T cells could fuse *in vitro* with skin-derived DCs (94), thus leading to multinucleated cell formation between T cells and DCs. Similarly, multinucleated giant HIV-1-infected macrophages have been found *in vivo* during infection in several different tissues, including lymph nodes, spleen, lungs, genital, and digestive tracts, and the central nervous system (CNS) (102–106, 108, 148, 150–153). While several groups showed the presence of infected multinucleated macrophages in tissues, and more specifically in the brain of HIV-1-infected patients and SIV-infected monkeys, the cellular and molecular mechanisms related to their formation remained poorly investigated, with only one *in vitro* study demonstrating a role for the HIV-1 auxiliary protein Nef in the formation of multinucleated macrophages (154).

We recently reported *in vitro* original results and proposed a model for the formation of HIV-1-infected MGCs related to a two-step cell–cell fusion process for HIV-1 cell-to-cell transfer from infected T cells and viral dissemination in macrophage targets (155) (Figure 1G). First, a heterotypic fusion occurs between HIV-1-infected T cells and macrophage targets for virus transfer. Then the newly formed lymphocyte/macrophage infected fused cells acquire the ability to fuse with surrounding uninfected macrophages for virus dissemination. As evidenced by cell imaging analyses, the first step is related to the establishment of contacts with infected T cells leading to the fusion of the infected T cells with macrophages. This cell fusion, dependent of the viral envelope/CD4 receptor interaction and restricted to CCR5-tropic viral strains, has been evidenced by a massive and fast transfer of Gag + material, as well as the presence of specific T cells markers (CD3, CD2, and Lck) in the cytoplasm and at the plasma membrane of the macrophage targets. The newly formed lymphocyte/macrophage fused cells (LMFCs) are then able to fuse with surrounding uninfected macrophages. These two sequential cell fusion processes are dependent on the viral envelope and lead to the formation of highly virus-productive MGCs that could survive for a long time in host tissues to produce infectious virus particles as shown *in vivo* in lymphoid organs and the CNS of HIV-1-infected patients and SIV-infected macaques (103, 106, 108, 152, 156). Similarly, the first step related to the initial T cell-to-macrophage fusion agrees with results showing that myeloid cells from lymphoid tissues of SIV-infected macaques contain T-cell markers and viral RNA and DNA originating from infected T cells (122, 123). This route of infection may be a major determinant *in vivo* for virus dissemination and establishment of macrophage virus reservoirs in host tissues.

## Conclusion

Since the HIV-1 discovery, a lot of works have been accomplished to decipher its life cycle, in order to counteract virus transmission and dissemination. Counteraction of host defenses is a major feature of HIV-1, since it is able to infect a large panel of immune cells and to largely decrease their effector functions. During the past years, several entry pathways for virus dissemination have been discovered but still need further investigations. While HIV-1 was initially suspected to enter its target cells only through fusion of viral particles with host T-cell membranes, it appears more and more evident that it can use a large diversity of cellular features to infect target cells, especially through direct cell-to-cell transmission by close contacts between an infected virus-donor cell and a target cell. Cell-to-cell transfer of HIV-1 indeed allows a massive release of viral material toward the target cell inducing a strong increase of viral infectivity, compared with infection with cell-free viruses. Depending on the infected donor cells as well as the target cell lineages, HIV-1 can take advantage of their specific physiology and features to infect its host. In T cell-to-T-cell transmission, HIV-1 provokes the formation of contacts, through Env–CD4 interactions, to allow a massive transfer of viruses between the two cells, thus improving its ability to enter the host cell. This intercellular structure, called virological synapse, is strongly similar to another but physiological one, the immunological synapse, indicating the HIV-1 ability to hijack T-cell pathways to spread. During trans-infection from DCs to target T cells, HIV-1 is even able to re-use at its advantage the interaction between APCs and T cells to cross the intercellular space for infection of target T cells. Through the interface formed between donor and target cells, filopodia coming from target T cells penetrate some VCCs in donor DCs to capture virions.

Therefore, intercellular communication pathways seem to be a road of choice for HIV-1 spreading. By increasing the frequency of filopodia formation in host infected cells, HIV-1 also enhances the surface of contacts of infected cells with the environment, thus improving its efficiency to be transferred to a non-infected neighboring target cell. In addition, HIV-1 can also create real bridges between cells, the so-called TNTs, which resemble to highways for virus transmission and dissemination. Moreover, when infected T cells are engulfed by specialized-phagocytic cells such macrophages, instead of being destroyed, HIV-1 is still able to infect the phagocyte.

Finally, HIV-1 uses its own ability to fuse with host cell membranes to provoke fusion between an infected donor cell and a target cell. By this way, HIV-1 is totally hidden form extracellular milieu since the sharing of the cytosol between the two cells appears to be sufficient for viral material dissemination and productive infection the new-formed fused cell able to produce new fully infectious virus particles. While fusion between T cells has been observed since a long time but was controverted as an *in vitro* artifact, more recent studies have pointed out the presence of small T-cell syncytia *in vivo* in lymph nodes from infected patients and proposed that these syncytia are fully able to transmit viral particles by cell-to-cell transfer to other cell targets. Multinucleated macrophages or DCs have also been observed *in vivo* in different tissues from HIV-1-infected patients, indicating the cell-to-cell fusogenicity of HIV-1 for different cell types. We have recently discovered a new mechanism of cell fusion between HIV-1-infected T cells and target macrophages. Heterotypic fusion between these cells appears, at least *in vitro*, to be a massive and very fast process allowing the formation of LMFCs that acquire the ability to fuse rapidly with neighboring non-infected macrophages, thus forming MGCs. The new-formed MGCs can survive for a long period *in vitro* and are highly productive of fully infectious viral particles. This important process could explain the presence of MGCs observed in several tissues of infected patients. Because of their long-time survival capacity, infected MGCs could participate in virus dissemination and establishment of persistent virus reservoirs in host tissues.

To conclude, while cell-associated HIV-1 transmission is believed to contribute to sexual transmission or viral spreading as well as to the formation of latent virus reservoirs, only few studies address the importance of cell-to-cell transfer of HIV-1 *in vivo*, and further investigations are needed to decipher the role of all the intercellular structures and processes described *in vitro* for pathogenesis in HIV-1-infected patients.

## Author Contributions

All authors listed, have made a substantial, direct, and intellectual contribution to the work, and approved it for publication.

## Conflict of Interest Statement

The authors declare that the research was conducted in the absence of any commercial or financial relationships that could be construed as a potential conflict of interest.
